# New Insights into Stroke from Continuous Passively Collected Temperature and Sleep Data Using Wrist-Worn Wearables

**DOI:** 10.3390/s23031069

**Published:** 2023-01-17

**Authors:** Katherine Edgley, Ho-Yan Yvonne Chun, William N. Whiteley, Athanasios Tsanas

**Affiliations:** 1 MRC Centre for Reproductive Health, University of Edinburgh, Edinburgh EH16 4TJ, UK; 2 Centre for Clinical Brain Sciences, University of Edinburgh, Edinburgh EH16 4SB, UK; 3 Usher Institute, Edinburgh Medical School, University of Edinburgh, Edinburgh EH16 4UX, UK; 4 Alan Turing Institute, London NW1 2DB, UK

**Keywords:** actigraphy, clinical decision support tool, sleep, stroke, wearable sensor

## Abstract

Actigraphy may provide new insights into clinical outcomes and symptom management of patients through passive, continuous data collection. We used the GENEActiv smartwatch to passively collect actigraphy, wrist temperature, and ambient light data from 27 participants after stroke or probable brain transient ischemic attack (TIA) over 42 periods of device wear. We computed 323 features using established algorithms and proposed 25 novel features to characterize sleep and temperature. We investigated statistical associations between the extracted features and clinical outcomes evaluated using clinically validated questionnaires to gain insight into post-stroke recovery. We subsequently fitted logistic regression models to replicate clinical diagnosis (stroke or TIA) and disability due to stroke. The model generalization performance was assessed using a leave-one-subject-out cross validation method with the selected feature subsets, reporting the area under the curve (AUC). We found that several novel features were strongly correlated (|r|>0.3) with stroke symptoms and mental health measures. Using selected novel features, we obtained an AUC of 0.766 to estimate diagnosis and an AUC of 0.749 to estimate whether disability due to stroke was present. Collectively, these findings suggest that features extracted from the temperature smartwatch sensor may reveal additional clinically useful information over and above existing actigraphy-based features.

## 1. Introduction

Many stroke survivors experience insomnia, nocturnal wakefulness, daytime sleepiness, and lower sleep efficiency [1]. Insomnia after stroke may hinder recovery [2]. However, stroke survivors are seldomly offered clinical sleep assessment [3], and previous work has reported that recovery care does not sufficiently address insomnia and sleep disorders, particularly in the chronic phase of stroke [1].

Ideally, stroke patients’ activity and sleep would be monitored and assessed longitudinally, to aid in the assessment of sleep as well as disability outcomes. This monitoring would additionally enable analysis of the interaction between stroke recovery, sleep, and activity to develop interventions and improve outcomes [4]. Furthermore, the evaluation of treatment efficacy can be aided through the use of longitudinal sleep data, as seen in other studies [5].

Polysomnography (PSG) is considered the “gold standard” of sleep assessment; however, it is invasive and requires the use of highly specialized and costly equipment, which precludes the possibility of scaling up to undertake long-term sleep assessment [6]. Actigraphy, a non-invasive technique collecting three-dimensional acceleration data, offers a practical solution towards providing insights into physical activity (PA) and sleep patterns over long periods of time through *passive* data collection. Actigraphy has previously demonstrated good agreement against PSG for those in normal adult populations, and it has also been shown effective for assessing sleep patterns for those with insomnia, particularly for evaluating the effect of treatment on sleep [7].

In actigraphy studies, participants typically have wrist-worn accelerometers, and acceleration from hand movement is continuously recorded for 24 h a day with a sufficiently high frequency (typically in the range from 1–100 Hz), depending on the device used and application. One can then extract different levels of PA and sleep onset and offset (wake) times from the raw actigraphy data, a more reliable method providing more information than using self-reporting sleep times alone [8]. Actigraphy data can, additionally, provide detailed information about the quality of sleep and awakening times [6].

Recent studies have demonstrated the potential for sleep, activity, and circadian variability assessment in providing clinically useful information in a variety of practical settings [5,9,10,11]. Sleep duration, sleep efficiency, and other characteristics of sleep are all influenced by the natural biological cycle, i.e., *circadian variability or rhythm* [12]. The cycle is influenced by external factors, such as light, but is also built into cell processes, influencing both core body temperature and sleep patterns [13]. Body temperature follows an approximately 24-h circadian rhythm, which has been found to generally decrease after getting into bed, potentially because of decreased activity, body metabolism, or in order to aid in the onset of sleep [14,15]. There is a lot of work on the role of *thermoregulation* (mechanism by which humans maintain their body temperature independently of ambient temperature) [16]. Temperature changes and temperature variability can provide insights into physiological aspects and pathophysiology, and, crucially for the purposes of this study, thermoregulation is affected by problems in the central nervous system and in sleep [17]. Studies have also found that waking from sleep generally occurs as body temperature rises [14].

Temperature sensors incorporated into wearable devices are commonly used for detecting non-wear times and calibrating accelerometers in long-term sleep and activity studies [18]. In this study, we used temperature readings from a standard commercial wearable sensor to extract new measures of daily variation and examine how they relate to clinical outcomes within a stroke cohort. Relatively few studies have aimed to extract additional information from the temperature readings to gain insight into patterns of individuals and differentiate between these patterns [19,20,21], although several studies have found that wrist temperature may be a useful proxy for evaluating sleep or circadian rhythms [22,23].

The aim of this study is to explore both known and novel measures extracted from passively recorded smartwatch data and to assess their utility towards providing new clinical insights into activity and sleep patterns of participants, using stroke as a testbed for our exploration.

## 2. Materials and Methods

### 2.1. Data

**Figure 1:**
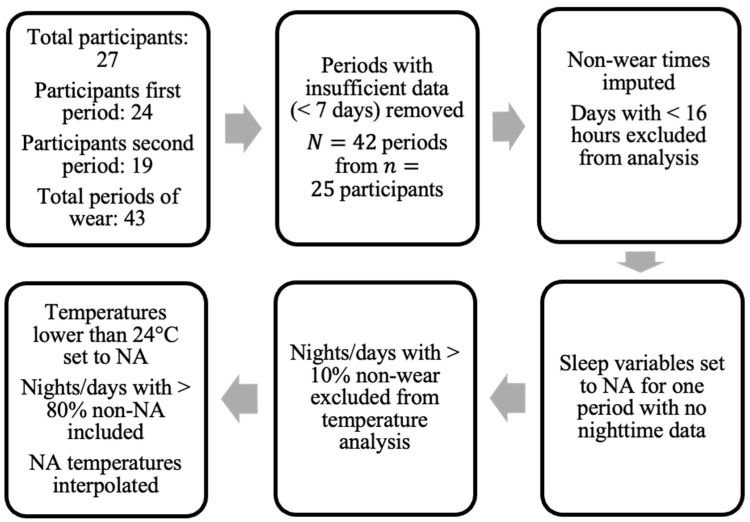
Flow-chart of data pre-processing. “Periods” denote separate treatment periods where data was collected (N=42) from n=25 participants in the study.

The data used in this study was collected by clinical colleagues at the Centre for Clinical Brain Sciences, University of Edinburgh, and was first reported in [5]. The dataset included both survey data and raw signal data from wrist-worn accelerometers. The aim of the overall project was to assess each stroke survivor over the course of two separate treatment periods, a relax treatment and a cognitive behavioral therapy (CBT)-based, guided self-help intervention. The devices were returned after each period of treatment (thus referred to herein as a “period of wear”). Figure 1 provides the overall flow-chart.

The survey data contained information for 27 participants, all of whom consented to also wearing the GENEActiv Original watches, (https://activinsights.com/technology/geneactiv, last accessed on 12 December 2022). The survey included baseline information such as sex, age, diagnosis, and survey assessments, which were also carried out after each period of wear. The questionnaires were completed using the Health Insurance Portability and Accountability Act of 1996 (HIPAA) compliant Research Electronic Data Capture (REDCap, version 7.1.2) application. Raw sensor data were provided for 24 of the 27 consenting participants for the first period of wear and 19 participants for the second period. We discarded data provided by participants if they did not collect at least seven valid days of data (with a valid day defined as >16 h of wear time), resulting in a sample size of 24 for the first period and 18 for the second period, totaling N=42 total periods of wear from n=25 participants (uppercase *N* is used to denote total sample size and lowercase *n* to denote the number of participants).

Excluding participants with insufficient sensor data (<7 days), 13 were male and 12 were female. Participant ages ranged from 39 to 81 years with (mean ± standard deviation) 64 ± 10 years. Of the 25 participants, the cohort comprised people who suffered from either ischemic stroke (14 participants), intracerebral hemorrhage (1 participant), or a probable brain transient ischemic attack (TIA) (10 participants). Participants were categorized by the clinical team into either the *stroke* group—ischemic stroke or intracerebral hemorrhage—or *TIA* group.

The survey assessments carried out at the baseline and after each period of wear included four different evaluations: (i) the modified Rankin Scale (mRS) evaluating disability [24,25], (ii) the Generalized Anxiety Disorder Questionnaire (GAD-7) evaluating anxiety [26], (iii) the 2-item Patient Health Questionnaire (PHQ-2) evaluating depression severity [27], and (iv) a modified version of the Fear Questionnaire (mFQ) evaluating phobias [28].

The mRS ranges from 0 (no symptoms) to 5 (severe disability) and is used to assess the level of disability in stroke patients (see [25]). A score of 3 or above indicates that a patient could not live alone without assistance.

The GAD-7 is used to assess the presence and severity of generalized anxiety disorder. The questionnaire involves seven questions, each with answers on a scale between zero and 3; the score on the questionnaire, thus, ranges between 0 and 21. It was suggested in [26] that a score from 5–10 may indicate mild anxiety, from 10–15 moderate anxiety, and over 15 severe anxiety.

The PHQ-2 involves two questions to establish levels of depression, each scored between 0 and 3, with a score of 3 or above suggested as a cut-off to screen for depression. This was posed as a brief alternative to the 9-item Patient Health Questionnaire (PHQ-9) and has been used for screening purposes [27].

Lastly, a modified version of the FQ was used to assess patients’ phobias. The questionnaire contains subscores for agoraphobia (FQ-ag), social phobia (FQ-soc), and specific phobia (FQ-sp). For each provided scenario, the patients ranked their avoidance on a scale from 0 (would not avoid) to 8 (always avoid). The subscores are derived from the relevant items on the questionnaire, with each subscore ranging from 0 to 40. Modifications were made to the FQ-specific phobia items as presented in [29], which involved replacement with alternatives relevant to stroke patients.

### 2.2. Feature Extraction from the Raw Signal Data

We used the GGIR package (version 1.9–2, configuration file available in Source Code S1 in the Supplementary Materials), maintained by van Hees for the public use of actigraphy processing tools, towards processing the raw actigraphy data from the binary form [30]. Before any further processing of the raw actigraphy data, it is critical to calibrate the outputs of the different devices; this is because intrinsically different three-dimensional accelerometer devices have different calibration offsets and they need to be aligned (this is both for the GENEActiv watches used in this study and other brands) [31]. This calibration of individual devices is typically achieved by either using axis-specific offsets for each of the three-dimensional data provided by the manufacturer [10], or identifying short signal segments of no movement, where, by definition, the magnitude of the acceleration should be equal to gravity (and, hence, we can correct accordingly and extrapolate across the entire recording for a device) [31]. From an implementation perspective, this is all part of the first step for the pre-processing of the data in the GGIR package used here, building on the work presented by van Hees et al. [10]. Based on the methods defined and rigorously analyzed in [30,31], features corresponding to the physical activity throughout the course of the participants’ accelerometer wearing were extracted.

The Euclidean norm minus one (ENMO) is a standard approach to summarize three-dimensional acceleration signals into a vector. Its extension, ENMO with negative values set to zero (ENMONZ) is often preferred and was used here within GGIR as a pre-processing step. ENMO is defined as the Euclidean norm of the three axes defined by the tri-axial accelerometer data with one gravitational unit subtracted, where ENMONZ indicates that negative ENMO values (due to inherent noise in the accelerometer sensor) have been set to zero. The effectiveness of using ENMONZ to summarize 3D acceleration has proved promising for differentiating sedentary behavior from “*motion-based light-intensity activities*”, with AUC > 0.95 [32].

The autocalibration process incorporated in the GGIR package was also employed for processing the raw actigraphy data, following the description by van Hees et al. [18]. Furthermore, as defined and implemented by GGIR, the actigraphy data were divided into 15-min blocks for the classification of non-wear periods, where the 60-min block centered at each 15-min block also informed the classification. To assign it as a non-wear period, the 60-min window must have a standard deviation of less than 13 mg or a value range of less than 50 mg for at least two of the three axes [30]. The process is further outlined in the supporting information of [31].

The pre-processing steps taken in this study regarding non-wear are presented in Figure 1. Only the days of patients where at least 16 h of wear time was detected were included in the analysis. Data for one patient in one period of treatment were excluded due to the lack of valid full days (<7 days). For non-wear periods on valid days, missing acceleration values were imputed by averaging values at a similar time on other days with available data (if available) [30].

In addition, when using temperature information extracted directly from the wrist-sensors, nights (defined by the GGIR parameter *fraction.night.invalid* as 24-h periods from 12 p.m. to 12 p.m.) with more than 10% of non-wear were excluded. Intervals where the accelerometer temperature reading dropped below 24 degrees Celsius were also imputed using linear interpolation, similarly to the method applied in [19] (where a threshold of 28 degrees was used). This threshold was chosen following visual inspection of temperature readings to exclude either short removals of the sensor or the influence of other external factors (e.g., cold water) on the sensor.

The GGIR package was also used to extract information about sleeping times and patterns of the patients [8,33]. No sleep diary was provided to the patients in this study, and, thus, the sleep detection algorithm proposed for actigraphy analysis without self-reported times was used, outlined in [33]. All sleep-related data were disregarded (set to NA) for one patient who consistently removed the wrist-sensor every night, since it would not be possible to assess nocturnal activity and sleep onset/offset. All information for wear periods when inferring average values per day was incorporated into the study, as all data were found to be informative for certain average measures, such as activity levels throughout the day.

These analyses of sleep and activity in the raw actigraphy data comprised part of the feature extraction process, where *features* are essentially characteristics of the raw data, ranging from sleep efficiency to manually defined measures or activity averages. Features automatically extracted through the GGIR package were subsequently examined, and three further features were computed based on those extracted from GGIR. This process resulted in extracting $M_{GGIR}=323$ features much larger than the number of participants (n=25), and samples when each wear time of the device (*period of wear*) was considered separately (N=42).

The following two sections describe the novel and temperature-based features proposed in this study, which complement the features extracted using the GGIR toolbox by van Hees et al.

### 2.3. Novel Sleep-Related Feature Extraction

In this study, we also propose new algorithms for deriving features, guided by medical intuition and related work in the field of actigraphy analysis. For convenience in referencing and to provide an overview of all features used in the study, we summarize them in Table 1 within their respective “feature groups” (algorithmic families). Approximate entropy (ApEn), a measure of the level of regularity in a time-series, was employed to extract features from temperature measurements taken by the accelerometer. ApEn has previously been used for extracting information from different types of time-series in biomedical applications [34].

*ApEn*, applied to a time-series $x=\{x_{1},\;x_{2},\;\ldots ,\;x_{K}\}$ of length *K* can be defined by the procedure presented in Table 2 in accordance with definitions in [35] with selected parameters *m* and *r*. For a given time-series ***x*** in our study, the proposed parameter of m=2 was used, and a value of r=0.2×sd(x) was selected [36].

The *weekend–weekday sleep duration difference* (WE–WD difference)*,* defined as the mean sleep duration over weekends minus the mean sleep duration over weekdays, aims to capture habits of sleeping disproportionately on weekends. A similar estimation of “social jet lag” has been carried out in [20] using mid-sleep points. The percentage of days per wear time of the device in which the participant sleeps before midnight, *onset before 00:00*, was also computed to capture healthy sleeping habits.

In addition, following visual inspection of the data, we observed that in certain patients, a clear upward or downward trend in several sleep-related variables was apparent over a single period of treatment. These changes were captured by the *trend of sleep variables* over the period of device wear, computed using the standardized best-fit regression line (ordinary least squares). We define $x=\{x_{1},\;x_{2},\;\ldots ,\;x_{nnight}\}$ as the series of *nnight* available nights for the period of wear (e.g., if the watch was not worn on day 1 the series would begin with 2); in addition, we define the series of the corresponding variable on each of these nights as $y=\left\{y_{1},\;y_{2},\;\ldots ,\;y_{nnight}\right\}$ (e.g., time of sleep onset for each available night). For each period of device wear, the Pearson correlation coefficient $\rho_{Pearson}\left(x,y\right)$ was computed. Using this method, which we referred to herein as the variable *trend,* was computed from the following variables to create six novel features: time of sleep onset and offset, duration of SPT-window and sustained activity bouts during the day (SIBD), number of sleep periods, and sleep efficiency.

### 2.4. Temperature-Based Feature Extraction

In addition to the sleep- and activity-based features, the temperature recording modality that the GENEActiv smartwatch records may provide useful information beyond calibration when used in feature extraction. Previous studies have investigated body temperature fluctuations in the process of feature extraction by assuming that a healthy participant will exhibit a decrease in body temperature that occurs after sleep onset and an increase in body temperature before waking [13,14]. However, circadian rhythm studies have found an inverse relationship between core temperature and distal skin temperature, and, thus, skin temperature can also be indicative of circadian rhythms, increasing during the sleep period [23]. With these temperature patterns in mind, features were computed with the aim of characterizing the extent to which a participant’s body temperature can be regarded as “healthy”, and in the following paragraph we describe our approach in further detail.

Temperature was averaged at 15-min intervals before extracting further information, as these features aimed to capture gradual temperature fluctuations. The temperature fluctuation recorded approximately every 15 min sufficed for feature extraction, which adheres to the Nyquist sampling theorem for our frequency of interest every 30 min [37]. The features derived from wrist temperature were extracted for each available sleep period within a valid night (less than 10% non-wear within the 24-h period from 12 p.m. to 12 p.m.)—determined using the automatically computed (through GGIR) time of sleep onset to offset, restricted to between 19:00 and 14:00 the following day—and day (defined as 08:00 to 08:00). A simple moving average was then applied over these time series to eliminate excess noise that may be present due to external factors. To compute the simple moving average with a rolling window size of *l* data points (where l=2q+1 denotes the entire length of the rolling window), each temperature at time point *j*, defined as $x_{j}$, was transformed by the following equation:

(1) $${\hat{x}}_{j}=\frac{\left(x_{j-q}+\ldots +x_{j-1}+x_{j}+x_{j+1}+\ldots +x_{j+q}\right)}{l}$$

For temperature feature extraction in this study, a value of *q =* 3 (l=7) was chosen based on visual observations of the noise elimination.

The simple moving average was, thus, computed over series of length 4×sleep duration (h) for each night, where sleep onset was rounded down and sleep offset rounded up to the nearest quarter hour. Over each full day, the simple moving average was computed over all 97 data points of the 24-h day. Temperature readings below 24 °C were imputed using linear interpolation (see Figure 1) prior to applying the moving average. After visual inspection of temperature readings, nights (19:00 to 14:00) or days (08:00 to 08:00) with fewer than 61 or 77 values (80% of all possible values) above this temperature threshold were excluded from feature extraction, due to the large number of missing values. Similarly, if fewer than 15 temperature data points were present between sleep onset and offset, the night was excluded.

The final extracted temperature features resulted from summarizing the data using standard statistical descriptors (mean and standard deviation) of temperature variables across all available days within a period of wear. Defining the time-series $x_{i}=\{x_{i,1},\;x_{i,2},\;\ldots ,\;x_{i,K}\}$ of temperature averaged at each of the *K* total points in time for a given night i (of nnight total nights), we extracted the following:

(2) $$T_{i}=mean(\{x_{i,\;\mathrm{onset}},\;\ldots ,\;x_{i,\;\mathrm{offset}})\},\;\mathrm{and}$$

(3) $$S_{i}=sd(\{x_{i,\;\mathrm{onset}},\;\ldots ,\;x_{i,\;\mathrm{offset}})\}.$$

We then computed the standard deviation of the mean temperature over available nights as $sd\left(\left\{T_{1},\;T_{2},\;\ldots ,\;T_{nnight}\right\}\right)$; similarly, we computed the mean and standard deviation of the standard deviation of temperature from sleep onset to offset as $sd\left(\left\{S_{1},\;S_{2},\;\ldots ,\;S_{nnight}\right\}\right)$ and $mean\left(\left\{S_{1},\;S_{2},\;\ldots ,\;S_{nnight}\right\}\right)$, respectively. These features provide information about temperature variation across sleeping times as well as an overview of the variability in wrist temperature throughout sleep.

Due to the influence of external factors that we have no control over, such as the tightness of the smartwatch on the wrist on the temperature recordings, the average temperature was not used directly as a feature. Instead, the standard deviation of the mean across all days was taken to capture major fluctuations of temperature between days. Similarly, the standard deviation of the *time of minimum and maximum temperatures over 24 h* was extracted to understand whether the minimum and maximum temperature generally occur at similar times of the day. These basic summary measures of temperature (mean, standard deviation, time of minimum and maximum) have been employed in a similar manner in other studies using wrist-worn temperature sensors [10,19].

Summary statistics (mean and standard deviation) were also taken for the $IQR\left(\left\{x_{i,\;\mathrm{onset}},\;\ldots ,\;x_{i,\;\mathrm{offset}}\right\}\right)$ over available nights of data, i.e., *interquartile range of temperature from sleep onset to offset,* as a second variability measure considering the variation in the middle 50% of the data. Mean and standard deviation of the *hours before waking/after sleep onset that temperature minimum and maximum occurs* was used to inform when the lowest/highest temperature took place relative to sleep onset, as body temperature is known to decrease substantially following the onset of sleep and increase close to the time of waking [38].

The change in temperature was also computed over each 15-min interval and used to calculate the maximum rate of increase and decrease during sleep. The time that the maximum rate of increase and decrease occurred was then computed, denoted as *time of MROI* and *time of MROD;* the mean and standard deviation of these variables were then extracted as features.

The *ApEn of temperature over 24 h and during sleep* was also used to capture variability in daily temperature changes and temperature changes from sleep onset to offset. Features were extracted for each period of wear by computing:

(4) $$ApEn(\{x_{i,1},\;\ldots ,\;x_{i,K}\})/K$$

for each given day or sleep period, i, and then computing both the mean and standard deviation over all available days or nights. ApEn values for each day or sleep period were normalized by dividing by the number of temperature points within the respective period, K, to avoid producing higher entropies for longer nights of sleep.

The ApEn of sleep temperature over all available nights (*nnight*) concatenated for each period of wear was also computed, denoted as *ApEn of sleep temp. (all nights)*. This was computed as:

(5) $$ApEn(\{x_{1,1},\ldots ,\;x_{1,K_{1}},\;\ldots ,\;x_{nnight,1},\ldots ,\;x_{nnight,K_{nnight}}\})/\left(K_{1}+\ldots +K_{nnight}\right),$$

where $K_{i}$ represents the number of total time points from sleep onset to offset for a given night i. Again, the ApEn value was divided by the total number of sample points as a normalization step.

Lastly, the difference between the mean temperature during sleep and during wake (*sleep–wake temp. diff.*) over a period of 48 h (starting at 00:00 on the day of sleep onset) was also computed, and then the mean and standard deviation of this measure were extracted as features. This concept was previously applied within [19], but we categorized it together with other temperature-based features.

### 2.5. Feature Pre-Processing

Using GGIR as well as engineering novel features, we extracted $M_{act}=$348 (323 known and 25 novel) features (where the subscript “act” denotes actigraphy features), a large number relative to the number of samples in the original data (N=42). As detailed in the following sections, we also regarded participant sex and age as features for the purpose of replicating diagnosis labels and stroke severity, thus, totaling M=350 features.

In this study, we aimed to assess the utility of the features in discriminating between stroke outcomes by mapping a selection of the extracted features onto (1) stroke severity as assessed by mRS after the period of device wear and (2) the diagnosis of patients (stroke or TIA). However, fitting a model with many features, without a correspondingly large number of samples, can lead to overfitting and, therefore, not yield accurate out-of-sample predictions [39]. Therefore, it was necessary to select a subset of the available features to estimate the outcomes at hand. The features were first categorized into the following groups: *demographic* (age and sex, i.e., 2 features), *sleep* (99 features), *PA* (224 features), and *novel* (25 features).

Ideally, one would select only the feature set that is jointly most predictive of the outcome and for discarding redundant or noisy features. Thus, to select only a small subset of features from each feature group that are jointly most effective in estimating stroke severity or diagnosis, we employed a feature selection algorithm called *relevance, redundancy, and complementarity trade-off* (RRCT) as proposed in [40]. RRCT builds upon previous algorithms that aim to maximize the feature relevance to the target variable while simultaneously minimizing the feature subset pairwise redundancies by using a correlation-based method. RRCT additionally considers interactions between features and their joint relevance to the target variable (complementarity) through partial correlation coefficients. The algorithm, when compared to other widely used feature selection algorithms, performs particularly well in datasets with a large number of features relative to the number of samples [40].

Prior to feature selection, one participant period of wear where sleep-related variables were masked, i.e., set to missing values, was removed entirely, resulting in a sample size of $N_{final}=41$ (“final” indicating the samples used for feature selection and classification) from n=25 participants. In addition, missing values were first imputed using the corresponding feature value for the *same* participant (thus, preventing any data leakage) where available. Finally, features with remaining missing values were removed, resulting in 10 PA features and 2 sleep features being omitted.

### 2.6. Exploratory Analysis

We used the design matrix of extracted features of size $N\times M_{act}$, where N=42 is the number of samples (each representing a period of wear) and $M_{act}=348$ is the number of GGIR and novel features, to investigate associations between these features and questionnaire outcomes (GAD-7, mFQ, PHQ, and mRS). To compare features with the questionnaire outcomes, the change in value of each questionnaire result was computed from the baseline (e.g., “mRS diff. from baseline”) and from the previous period of device wear (e.g., “mRS change”). Additionally, the questionnaire value after each period of wear was extracted (e.g., “mRS after”). As PHQ-2 was recorded only at the baseline and after the first period of wear, both of these values were extracted without modification.

To measure the statistical association between derived features and questionnaire outcomes, correlation analysis was used to quantify the extent of the statistical relationships. We used the *Spearman correlation coefficient*, which quantifies the extent of monotonic relationships, as opposed to solely linear relationships (quantified using the Pearson correlation coefficient). Spearman correlation was also used for ordinal categorical variables (taking values on a scale). Correlations were considered to be *statistically strong* where |r|  > 0.3, and the level of α=0.05 was used to assess whether the correlations were *statistically significant* [41].

### 2.7. Feature Selection

After the additional feature pre-processing outlined in Section 2.5, the feature selection process was applied using a leave-one-subject-out cross validation (CV) method, equivalent to the method employed to evaluate out-of-sample accuracy of models fit to estimate diagnosis and stroke severity (see Section 2.8). The period of wear with no sleep-related data was removed at this stage, resulting in $N_{final}=41$ periods of wear used. In each iteration of feature selection, periods of device wear from a single participant were excluded, and all remaining samples were used to select features via RRCT. This resulted in a matrix of size $n\times M_{sel}$, where n=25 iterations and $M_{sel}$ represents the number of selected features, appearing in descending order of “importance”, i.e., in order of selection by the RRCT algorithm.

From a practical perspective, the feature order for perturbed versions of a dataset (e.g., as a result of using bootstrapping or having removed samples e.g., in a CV scheme) will lead to a different order for the ranked features. Therefore, we need to develop a strategy to determine the order of selected features so that it is consistently applied. To select the desired $M_{sel}$ features from the 25 repetitions we relied on the “voting scheme” proposed in [42] (for the implementation of the algorithm see “Voting mechanism for feature selection” in https://www.darth-group.com/software, last accessed on 12 December 2022). In this method, for a given feature number i of $M_{sel}$ total features (in descending order of importance), the feature occurring most frequently in the first i columns was added to the final set, if not already selected. In case of a tie, the feature with the lower index was selected [42]. To examine which features contributed most in each category, the top 10 features were selected using the voting method from the following feature groups separately: sleep, PA, novel, and all features combined. In addition, a “stability score” was computed for each of the top 10 features selected using the voting scheme. This score was defined as the percentage of cross-validation folds in which the feature appeared. To control for demographic variables, sex and age were additionally added to the sleep, PA, and novel feature groups prior to feature selection.

### 2.8. Estimating Stroke Severity and Diagnosis Using Feature Subsets

To understand the contribution of differing feature groups as categorized in Table 1—sleep, PA, and novel features—towards the estimation of stroke severity (mRS) and diagnosis, for each outcome we generated separate models for each feature group (using the selected features from each group), and one model for the selected features from the combined feature groups.

To assess the usefulness of selected features in estimating stroke severity as measured by mRS, we first aimed to map the features selected using RRCT in each feature group onto discretized mRS outcomes. Within this stroke cohort (after the removal of periods with insufficient wear), the majority of mRS scores after a period of device wear were either 0 ($N_{0}=14)$ or 1 ($N_{1}=18)$, while the remainder ($N_{\geq 2}=9)$ had an mRS from 2 to 4. Due to the sparsity of mRS scores above 1, to estimate stroke severity, we categorized the mRS outcome into two groups, mRS of 0 or above 0. For convenience, these are henceforth referred to as the *stroke severity groups* in this study. This discretization method was chosen due to the clinical relevance of comparing participants with no stroke disability to those with any disability.

Similarly, we aimed to map selected features in each feature group to the diagnosis of participants. Through the estimation of patient diagnosis, we aimed to assess the importance of different features towards characterizing the sleep and circadian rhythms of the two diagnosis groups. As two classes of diagnosis were present—probable brain TIA and stroke (ischemic stroke or intracerebral hemorrhage)—as well as two classes of stroke severity group, *binary logistic regression* models were used to classify patients aiming to replicate the clinical labels.

Logistic regression models with L2 penalization (with model parameters as defined in Source Code S1 in the Supplementary Materials) were fit to replicate stroke severity and diagnosis using the top 10 features selected using RRCT for each feature group detailed in Table 1 and for all features combined. Within each subset of selected features (sleep, PA, novel, and combined features), features were incrementally added to the model; thus, the first model was fit using only the top feature, the second with the top two features, etc. A threshold of 10 features was chosen due to the small number of samples available ($N_{final}=41$) to avoid separation, i.e., when features “perfectly separate” the binary target variable [43], which was found to occur when including 9 or more features in the logistic regression model.

When fitting the models, the mean and standard deviation for each feature within the training set were used to standardize both the training and test set. As previously stated, sex and age were included in each feature group prior to feature selection using RRCT to control for the demographics of participants; sex and age were, thus, selected as “relevant” features in some subsets.

To assess whether these classifiers will perform well on new, unseen data, the out-of-sample performance of these models was estimated using leave-one-subject-out CV, where in each iteration, samples from a *single participant* were held out. That is, in each iteration, samples from one participant were held out as a test set, and a model was fit on samples from the remaining participants (thus, avoiding contaminating the test set with samples from a participant that has been used to train the statistical learning model). This approach ensured that no information from the hold-out samples was present in the training set. The resulting performance of these models was presented by reporting the area under the receiver operating characteristic (ROC) curve (AUC), as well as balanced accuracy (when estimating the stroke severity group) because the dataset was unbalanced. Due to the deterministic nature of the leave-one-subject-out CV approach, the performance is consistent across CV repetitions and, thus, no range of performance was reported.

### 2.9. Estimating Stroke Severity and Diagnosis by Combining Feature Subsets

After logistic regression models were fit to replicate stroke severity groups and patient diagnosis (stroke or TIA) using the selected feature subsets (sleep, PA, novel, combined features), we aimed to combine the sleep, PA, novel, and combined subsets into a single model for each outcome. To do this we relied upon an approach where outputs of the four models were combined using a weighted average, known as a *weighted average ensemble*. First, the number of features that maximized out-of-sample AUC were selected for each feature group. These “maximum” AUCs were then used to weight the final probabilistic outputs from each separate logistic regression model as follows. Defining $p_{i,j}$ as the probabilistic output for sample i from the model fit on feature subset *j,* and defining $w_{j}$ as the maximum AUC for feature subset *j*, the final weighted average for a given sample i was computed as:

(6) $${\hat{p}}_{i}=\frac{w_{1}p_{i,1}+w_{2}p_{i,2}+w_{3}p_{i,3}+w_{4}p_{i,4}}{\sum w_{j}}$$

Using this weighted average, a final probabilistic output was generated for each sample in the data, and for each outcome (diagnosis and stroke severity group), and the resulting outputs were used to compute the performance of the models.

## 3. Results

### 3.1. Exploratory Analysis

**Figure 2:**
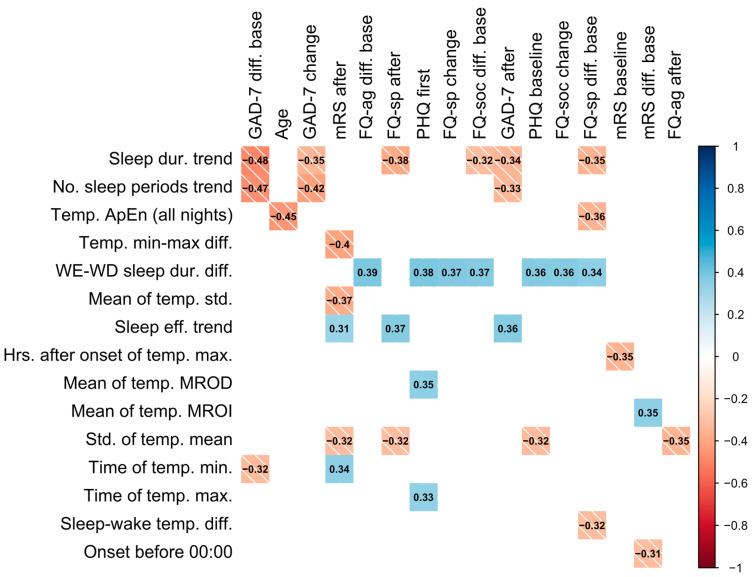
Colored correlation matrix indicating statistically significant (*p* < 0.05) Spearman correlations between novel features and questionnaire results, where each variable was computed over a single period of device wear. Only novel features and outcomes with statistically significant correlations are included in the plot. The shade of the square represents the direction of the relationship. “After” denotes the value after a period (of device wear), “change” denotes the change from the previous period, and “diff. base” denotes the change from baseline assessment.

Statistically strong correlations between questionnaire results and novel features are presented in Figure 2. These correlations suggest that, firstly, an increase in number of sleep periods (*no. sleep periods trend*) and sleep duration (*sleep dur. trend*) over a period of device wear is correlated with a decrease in GAD-7 scores relative to the baseline and previous score, as well as with a lower GAD-7 score after a period of wear. This may reflect that an increase in sleep duration (positively correlated with number of sleep periods) corresponds to a decrease in GAD-7 score, indicating an improvement in anxiety. Similarly, increases in sleep duration across a period of wear was associated with lower specific phobias.

A higher variation in temperature (*std. of temp. mean*) was associated with an improvement in stroke symptoms as measured by mRS and was also found to be higher in patients with a TIA diagnosis (0.820 ± 0.202) compared to those with stroke (0.725 ± 0.193). In addition, the earlier the time of the minimum temperature (*time of temp. min.)* was found to occur was correlated with a lower value of mRS (less severe symptoms). Similarly, sleeping before midnight (*onset before 00:00)* was correlated with a decrease in the mRS from the baseline. Participants sleeping longer on weekends compared to weekdays (*weekend–weekday sleep duration difference*) were also found to have higher questionnaire results and less improvement in specific phobias (FQ-sp) (see Figure 2). Age was also negatively correlated with the ApEn of sleep temperature (*ApEn sleep temp. (all nights)*) and the mean difference between sleep and wake temperature (*mean temp. sleep–wake diff.*).

### 3.2. Replicating Diagnosis Labels

Table 3 (upper section) outlines the subsets of top 10 features selected from each feature group using RRCT towards estimating diagnosis. Models were fit using these feature subsets by incrementally adding in features in descending order of importance, and out-of-sample performance was estimated using leave-one-subject-out CV (as outlined in Section 2.8). The “stability score” was computed as the number of CV iterations in which each feature was selected in the top 10; we found that although several features were selected in almost every iteration, the selection, overall, tended to vary moderately.

**Figure 3:**
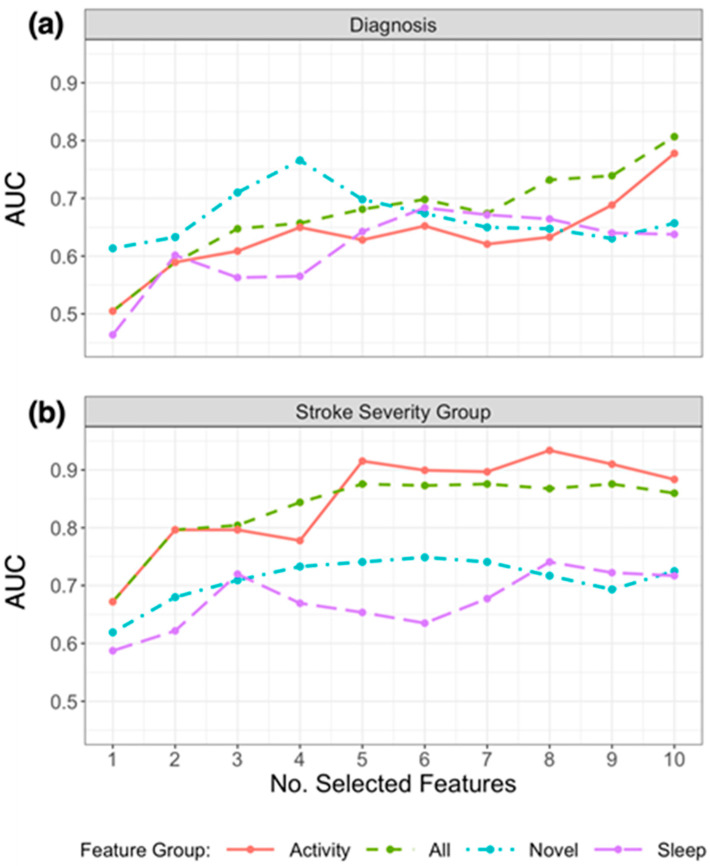
Plot of AUC results for replicating (**a**) diagnosis labels and (**b**) stroke severity group using logistic regression, by the number of selected features chosen using RRCT in descending order of importance. Feature groups are as defined in the first column of Table 1, and the top 10 selected features from each group are presented in Table 3. The horizontal axis indicates the number of selected features presented into the statistical learner, in descending order of selection as determined using RRCT (i.e., the first feature is the top choice of RRCT, the second feature is the second choice etc.).

**Figure 4:**
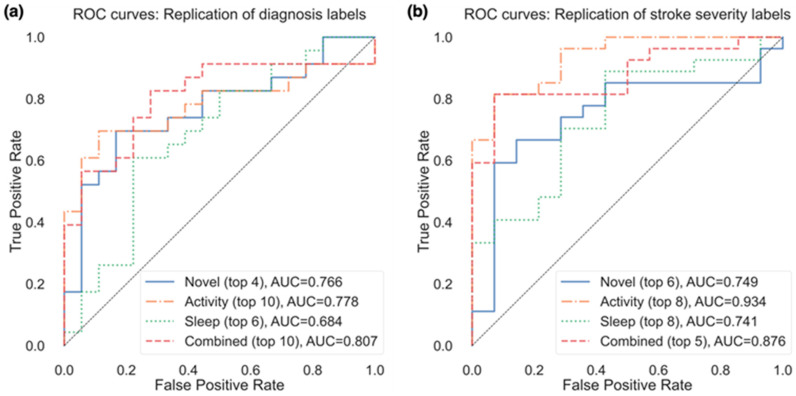
ROC curves for the replication of both (**a**) diagnosis labels and (**b**) stroke severity group, using the top features from each feature group resulting in the best AUC (as shown in parentheses after each feature group).

Due to the small sample size and validation method, the performance of models to classify patients varied considerably depending on the number of the selected features. Figure 3a presents the resulting AUC based on the number of selected features from the subsets in Table 3 that were used in the model, and Figure 4a presents the ROC curves for the top features (resulting in highest AUC) within each feature group. Using the top 4 selected novel features to classify diagnosis of participants resulted in an AUC of 0.766, using the top 10 PA features resulted in an AUC of 0.778, and the top 6 sleep features an AUC of 0.684. When RRCT was used to select from all features combined, the top 10 selected features resulted in the highest AUC of 0.807.

We also used the top 4 novel features, top 10 PA features, top 6 sleep features, and top 10 from all features combined to create the weighted model outlined in Section 2.9. For each iteration of leave-one-subject-out CV, four probabilistic outputs (one from each model) were generated for each of the held-out participant samples. To generate the final model output, a weighted average of the four individual model outputs was computed; weights corresponded to the maximum AUC achieved by the individual model. This weighted average ensemble model had improved performance (AUC of 0.891) compared to the model using a subset of all features combined (AUC of 0.807).

### 3.3. Replicating Stroke Severity Labels

Table 3 (lower section) outlines the subsets of the top 10 features selected from each feature group using RRCT towards estimating the stroke severity group, and Figure 4b presents the ROC curves for the top features (resulting in the highest AUC) within each feature group. As for diagnosis, the stability scores presented demonstrate that there was some variation in the features selected in each iteration (ranging from 32% to 100%). Figure 3b presents the resulting AUC based on the number of selected features from the subsets in Table 3 that were used in the model.

**Figure 5:**
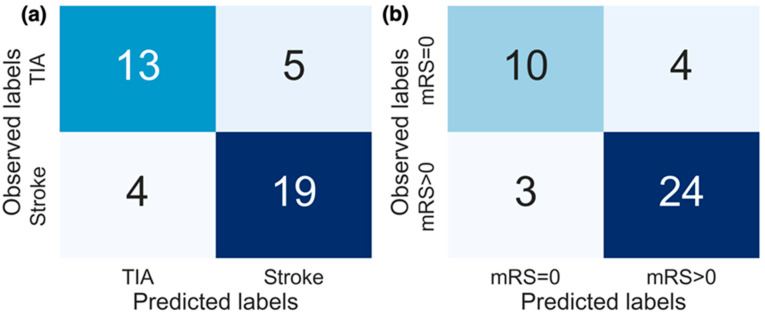
Confusion matrices for the replication of both (**a**) diagnosis labels and (**b**) stroke severity group. The results from models producing the best performance (AUC) are displayed. For replicating diagnosis, the top 10 features selected from all features combined were used, and for replicating stroke severity group the top 8 selected activity features were used.

Of the actigraphy-extracted features, PA-related features performed the best when estimating whether disability due to stroke was present. Using the top 8 PA features resulted in an AUC of 0.934 (confusion matrix shown in Figure 5b with a balanced accuracy of 0.802), and using the top 6 novel features, the model achieved an AUC of 0.749 (balanced accuracy of 0.676).

In the model containing a selected subset from all features combined, the model containing the top 5 selected features resulted in the highest AUC of 0.876, with a balanced accuracy of 0.676 (while the top 6 features resulted in a higher balanced accuracy of 0.766). The weighted model resulted in an AUC of 0.915, with a balanced accuracy of 0.749.

## 4. Discussion

This study investigated the application and development of actigraphic features to provide new insights into activity and sleep patterns in a cohort of stroke patients. The study demonstrated the potential of wrist-recorded temperature-based features to gain further insight into patients’ activities and sleep. In our study, several temperature-based features were shown to convey clinically useful information towards discriminating between patients that suffered from an ischemic stroke (or intracerebral hemorrhage) from those with a probable brain TIA. Using the novel features presented in this study, we were able to replicate patient diagnosis labels (stroke or TIA) with an AUC of 0.766. By combining models fit on previously used actigraphy features with these novel features in a weighted model, the performance increased to an AUC of 0.891. In addition, other novel and temperature-based features were found to be strongly correlated with age, anxiety and depression measures (GAD-7, PHQ, modified FQ), and stroke symptoms measured by mRS as illustrated in Figure 2. Temperature-based features were not found to be as accurate in estimating the stroke severity group as PA features, however, as shown in Figure 3b.

The temperature- and sleep-based features described in Section 2.3 and Section 2.4 have not been previously assessed in a cohort of stroke patients. However, some of the basic features included in this study have appeared in previous studies. In particular, the mean and standard deviation of wrist surface temperature, as well as the difference between sleep and wake temperature, were extracted in [19] and compared between groups by age and condition. In addition, the common approach of quantifying minimum and maximum temperatures (and corresponding times) and temperature range has also been used [10]. In summary, the completely novel features extracted in this study were WE–WD difference; onset before 00:00; trend of sleep variables; hours before waking/after sleep onset that temperature maximum and minimum occurred; time of MROI; time of MROD; ApEn of temperature over 24 h and during sleep; ApEn of sleep across all nights. To our knowledge, these features have not been previously used in actigraphy studies.

Investigating temperature throughout the day as well as during sleep, we found that several temperature-based features may aid in providing insight into the behavior or environment of patients. Notably, the ApEn of temperature during sleep, the time that the temperature minimum occurred, and the standard deviation of the temperature mean were found to have statistically strong correlations with questionnaire outcomes (Figure 2), particularly with mRS values in the case of the latter two features. These temperature-based features may reveal clinically useful information about changes in environment day-to-day, such as going outdoors. For instance, the minimum temperature throughout the day may correspond to time spent outdoors, although we do not have additional external evidence (e.g., from a wearable camera) to verify this plausible hypothesis.

The difference observed in the standard deviation of mean temperature between diagnoses may also relate to variation in the external environment, for instance, the room temperature or the frequency that someone goes outdoors. As this measurement was lower in stroke patients (Section 3.1), we believe that the stroke patients may experience less variation in environment and time spent outdoors. We also found evidence of a statistically strong association between phobias (modified FQ) and the difference in sleep duration between weekends and weekdays, suggesting a link between less regular sleep patterns (i.e., sleeping considerably more on weekends compared to weekdays) and increased anxiety and phobia.

The presence of a large number of features in the dataset, and the fact that many of the features are pairwise statistically strongly associated (see Supplementary Table S1 in the Supplementary Materials) motivates the use of feature selection to ensure we obtain a compact feature subset and a parsimonious statistical learning model [44]. In this study, we used RRCT, a robust and powerful feature selection algorithm we proposed and extensively validated recently, which is particularly suitable for datasets with small sample sizes [40]. When assessing the out-of-sample performance of models used to replicate patient diagnosis labels (stroke or TIA), we found that using a selected subset of novel features extracted in this study alone, the logistic regression model could estimate the diagnosis of participants with an AUC of 0.766. In addition, several temperature-based features were selected in the top 10 features using RRCT from all features combined when replicating both diagnosis and stroke severity, which strongly supports the notion that there is clinically useful information in temperature data over and above actigraphy. The performance of these models aiming to replicate diagnosis labels provides further evidence of the potential to use temperature-based features to gain insight into behavioral patterns that may be clinically useful.

Due to the limited sample size of the dataset, we are pragmatically restricted in the choice of statistical learners we can use: it is well-known that advanced nonlinear statistical learners (e.g., support vector machines, tree-based approaches such as random forests) require a very large number of samples to adequately explore the feature space and build an accurate predictive model [44]. Therefore, we only explored other simple statistical learning methods to compare against the findings presented using logistic regression. Specifically, we used linear discriminant analysis and naïve bayes classifiers for the same tasks as using logistic regression. The results were similar to or worse than what we found with logistic regression (naïve bayes: best AUC of 0.630 for replicating diagnosis and 0.772 for replicating the stroke severity group; linear discriminant analysis: best AUC of 0.785 for replicating diagnosis and 0.926 for replicating the stroke severity group) and, hence, were not reported in detail in this study for brevity. Because of the sample size limitation, we have similarly resorted to largely default parameter values for the statistical learners used and did not explore parameter optimization methods.

We note that baseline mRS was found to be highly correlated to mRS after treatment ($\rho_{Spearman}=0.636$), and, therefore, baseline mRS would provide a good indication of mRS after treatment in this small dataset. However, as we aimed to estimate the stroke severity group of patients using actigraphy features alone, we did not incorporate baseline questionnaire scores in the models. For that reason, propagating the baseline mRS score as an outcome and comparing that naïve benchmark model against the statistical learning model developed here would not be a fair comparison against the developed algorithm (given that the model does not have any baseline information or knowledge about specific participants other than the actigraphy-based features extracted).

There are different methodological strategies towards selecting a robust feature subset. One approach is to use nested cross-validation where the inner loop acts as a feature selector and the outer loop is used to assess the statistical learning model’s performance. However, fundamentally this approach may be very sensitive to statistical data fluctuations due to the samples used in each CV fold, particularly in studies with limited sample sizes. Moreover, from a practical perspective, there will typically be different feature sets that are selected in the CV folds, and it is not clear which minimal feature set that we should be using (depending on the setting this may have implications for the features collected or extracted) to minimize computation cost. In this approach, we could subsequently apply feature selection on the entire dataset to determine a single feature subset that will be reported for further use in future applications, however, that is not the feature set that was used to report out-of-sample findings. This has motivated the development of an alternative approach where the feature selection and the statistical mapping process are not nested, which we have developed and validated across a range of problems and datasets [40,42,45,46,47,48,49]. Arguably, the practical aim of feature selection is to determine a stable parsimonious feature set that is jointly highly predictive of the response in combination with a statistical learner (in some practical applications, additional considerations such as economical cost towards collecting certain features may be worth exploring, e.g., see [50]). The major advantage of this second approach (which we also used in this study) is that we determine a consistent feature set, which is subsequently used across *all* iterations in the statistical learning process towards assessing model performance, and, hence, reflects the model that would be put forward for external validation. For further background, including the philosophical context of this second approach, arguments and counter-arguments for its use, we refer readers to [40,42,46,49]. We acknowledge that similarly to many competing methodologies in data analytics there are often pros and cons when comparing competing approaches, and this is an area that deserves further theoretical methodological investigation.

Relatively few studies have previously explored the use of the temperature modality in wrist-worn wearable sensors to extract features that could be used to estimate clinical outcomes. In this paper, we presented novel work and demonstrated the potential utility of wearable temperature sensors by extracting information beyond summary statistics to include more complex information from wrist temperature fluctuations. A previous study utilized the temperature sensor from a custom-built wristband to extract summary statistics (mean and standard deviation) of temperature over 24-h days during sleep and wake periods as well as parametric and non-parametric measures of circadian rhythm (temperature statistics, inter-day stability, intra-day variability, and Cosinor model parameters) [19]. The authors compared the above-mentioned features between younger adults, older adults, and older adults with dementia, reporting lower differences between sleep and wake temperature as well as lower inter-daily stability of temperature rhythms in adults with dementia. In our study, a similar decrease in the sleep–wake temperature difference was found among patients with stroke compared to those with TIA, although this was not selected as one of the top 10 features (shown in Table 3). In addition, the significantly lower amplitude of temperature rhythms found in adults with dementia bears similarity to our study, where a lower standard deviation in temperature mean was found among stroke patients. That study also found lower intra-daily variability values among older adults, potentially suggesting increased sleep fragmentation or daytime sleep [19]. The ApEn of sleep temperature extracted in our study, which was elevated among stroke patients, may similarly reveal more disturbed sleep. In another study, basic temperature features such as the minimum/maximum temperature and associated time were extracted from temperature sensors in GENEActiv wrist sensors and compared between patients with post-traumatic stress disorder (PTSD) and control groups in [10]. However, these temperature features were not reported as statistically significantly different between groups in that study.

The novel features presented in this paper may be useful for extending analyses relying on actigraphy data as well as for providing further insight into the relationship between post-stroke symptoms, sleep, and physical activity. The primary limitation of the study is the cohort size and the number of available samples. We note that the use of actigraphy in this patient group has not been previously explored in detail and, therefore, the reported results should be viewed as early proof-of-concept findings that need to be further explored in a larger dataset. Many aspects of this study could be expanded and investigated in further depth. The sample size and extent of the data provided limits not only the possible algorithms that can be applied to the dataset but also the statistical confidence in the presented findings, which need to be interpreted tentatively. A more extensive dataset would provide more robust results and statistical associations could be verified with sufficient statistical power. Furthermore, self-reported sleep onset and offset times along with self-reported assessment of awakenings and sleep quality would provide additional insight into differences between perceived sleep and actigraphic estimates of sleep.

The study may also benefit from more complex sleep and circadian rhythm feature extraction, which could allow for an analysis of the extent to which wrist temperature improves the understanding of these circadian rhythms. Additionally, building on the insights from temperature-based features presented in this study, we recommend further examination of the effect of environment on wrist temperature in a larger dataset with recorded times of indoor versus outdoor activity to test this hypothesis, as actigraphy studies would benefit from further information about time spent outdoors as it relates to health and wellbeing.

In future work, we aim to assess the generalizability of the novel features presented in this paper in additional datasets, as well as assess the performance of these features using additional statistical learning methods. Furthermore, we plan to develop further algorithms applied to temperature data from wrist-worn wearables to shed light on the circadian rhythms of individuals, building on promising findings reported previously [19].

## 5. Conclusions

Modern wrist-worn devices have embedded diverse sensors that often provide high resolution recordings of modalities, such as three-dimensional acceleration, wrist temperature, and ambient light. This study makes a strong case that the wrist-recorded temperature modality, which, hitherto, has not been systematically investigated in the research literature, may contain clinically useful information. We demonstrated that the novel features derived from temperature and sleep proposed in this study provide clinically useful insights into stroke assessments regarding behaviors or activities that are not captured by actigraphy data alone. The current study’s findings are compelling and show potential towards further exploring the association of temperature with anxiety, fear, and depression. It is likely there is considerable potential for future work to explore information fusion approaches towards jointly processing wrist-recorded data modalities, such as three-dimensional acceleration and temperature data, to develop more robust clinical decision support tools. However, due to the limited statistical power in the stroke cohort available in this study, our findings should be treated tentatively and further work is warranted to establish how well these generalize across larger cohorts and different pathologies where multimodal wrist-worn data are collected.

## Figures and Tables

**Table 1:** Summary of feature groups used in this study. Total movement and duration of movement during the SPT-window and percent of activity at night were computed using GGIR-extracted features, rather than extracted directly. We computed 323 features using known methods and propose 25 novel features to characterize sleep and temperature in this study.

Feature Group	Type	Brief Description	Number of Features
Sleep	Basic sleep	Summary statistics of sleep duration, time of sleep onset and offset, sleep period time window (SPT-window)	58
	Sleep efficiency	Ratio of time sleeping to time spent in bed	8
	Night-waking	Movement or duration of waking during the SPT-window, percent of activity at night	31
	Circadian rhythm	Inter-daily stability and intra-daily variability	2
Physical Activity	Activity	Light activity bouts (LIGB), blocks of vigorous activity or moderate-to-vigorous physical activity (MVPA)	169
	Sustained inactivity bouts (SIB)	Inactivity bouts not during sleep period and SIBD	27
	L5 and M5 h	Five hours of day with highest (M5) and lowest (L5) acceleration	28
Novel	Sleep	WE–WD difference, onset before 12 a.m., trend in sleep variables	6
	Temperature	Temp. during sleep: mean, std., ApEn, max. rate of increase/decrease, h after onset of max/min. Temp. over 24 h: min, max, ApEn, min-max difference. Sleep–wake temp. diff.	19

**Table 2:** Definition of approximate Entropy (*ApEn*) as proposed in [35]. We used parameter values of m=2 and r=0.2×sd(x) as recommended in [36].

$ApEn(m,r,k)\;\mathrm{applied}\;\mathrm{to}\;a\text{ time-series }x=\{x_{1},\;x_{2},\;\ldots ,\;x_{K}\}$
$\mathrm{Define}\;a\;\mathrm{vector}\;\mathrm{of}\;\mathrm{length}\;m\;\mathrm{that}\;\mathrm{begins}\;\mathrm{with}\;x_{i},\;\mathrm{as}\;u_{i}=\left\{x_{i},\;x_{i+1},\;\ldots ,\;x_{i+m-1}\right\},\;\mathrm{where}\;1\leq i\;\leq K-m+1.\;\mathrm{Then},\;\mathrm{form}\;a\;$ $\mathrm{sequence}\;\mathrm{of}\;\mathrm{these}\;\mathrm{vectors}\;\mathrm{that}\;\mathrm{covers}\;\mathrm{all}\;\mathrm{points}\;\mathrm{in}\;\mathrm{the}\;\mathrm{time}\;\mathrm{series}:\;\left\{u_{1},\;u_{2},\;\ldots ,\;u_{K-m+1}\right\}$
$\mathrm{Define}\;\mathrm{the}\;\mathrm{distance}\;d\;\mathrm{between}\;u_{i}\;\mathrm{and}\;u_{j}\;\mathrm{as}\;d\left[u_{i},\;u_{j}\right]=\underset{1\leq l\leq m}{\max}\left|x_{i+l-1}-x_{j+l-1}\right|$
$\mathrm{Define}\;C_{i}^{m}\left(r\right)=\left(\mathrm{number}\;\mathrm{of}\;u_{j}\;\mathrm{such}\;\mathrm{that}\;d\left[u_{i},\;u_{j}\right]\leq \;r\right)/\left(K-m+1\right)\;,\;\mathrm{where}\;1\leq i\leq K-m+1$
$\mathrm{The}\;\mathrm{scalar}\;\mathrm{defined}\;\mathrm{as}\;\emptyset^{m}\left(r\right)=\frac{1}{K-m+1}\overset{K-m+1}{\underset{i=1}{\sum }}\log C_{i}^{m}\left(r\right)\;\mathrm{then}\;\mathrm{gives}:\;ApEn\left(m,r,K\right)=\left[\emptyset^{m}\left(r\right)-\emptyset^{m+1}\left(r\right)\right]$

**Table 3:** The subset of the top 10 selected features using RRCT are presented for estimating diagnosis (upper section) and stroke severity group (lower section) for the respective feature groups (defined by vertical labels). Adjacent to each feature the “stability score” (%) for that feature is presented. Demographics were included in all feature groups prior to feature selection to account for their potential effect on model performance. WD and WE denote weekdays and weekends, respectively. IN denotes inactivity, LIG denotes light activity, MOD denotes moderate activity, VIG denotes vigorous activity, and MVPA denotes moderate to vigorous activity; “wei” indicates a weighted average across available days, with weekend days weighted 2/5 weekdays.

	Sleep	%	Physical Activity	%	Novel	%	Combined	%
**Diagnosis** **(TIA or stroke)**	Sex	92	MVPA movement	96	ApEn of sleep temp.	100	MVPA movement	96
	Nightwake LIG movement	76	Sex	100	Hrs. before wake of temp. min.	88	Sex	92
	Std. of sleep eff. (WE)	88	Mean dur. SIBD (WE)	36	Sex	96	Nightwake MOD movement	64
	Dur. nightwake VIG (wei)	84	LIGB movement (wei)	84	Time of temp. min.	72	Hrs. before wake of temp. min.	44
	Std. no. sleep periods (WE)	48	Mean dur. SIBD	32	WE–WD sleep difference	92	LIG movement (wei)	80
	Mean SPT duration (WD)	40	Std. of SIBD dur.	48	Perc. Onset before 00:00	56	ApEn of sleep temp.	80
	Intra-daily variability	80	Age	92	Sleep eff. Trend	60	Age	68
	Age	68	Std. of SIBD dur. (WD)	32	Std. of temp. mean	44	Mean dur. SIBD	36
	Nightwake IN movement (wei)	64	Mean no. SIBD	44	Std. of temp. MROI	52	Movement in L5 h (WE)	44
	Std. of sleep onset	48	Mean dur. VIG	88	Std. of temp. MROD	52	Mean dur. SIBD (wei)	52
**Stroke Severity (0 ≥ 1)**	Mean no. sleep bouts (WD)	76	Mean dur. SIB	92	Age	100	Dur. SIB	92
	Std. of sleep onset (WD)	88	Age	100	Temp. min–max diff.	72	Age	88
	Age	100	Sex	80	SIBD trend	100	Nightwake MOD movement (wei)	68
	Sex	88	Movement IN	88	Mean time of temp. MROI	48	Mean movement (WD)	52
	Intra-daily variability	68	Movement IN (wei)	80	No. sleep periods trend	68	SIBD trend	56
	Std. of SPT dur. (WE)	72	Movement in L5 h (WE)	64	WE–WD sleep difference	88	Std. of SIBD dur. (WD)	52
	Mean sleep dur. (WE)	56	Mean movement (WE)	52	Temp. sleep–wake diff.	48	IN movement	44
	Nightwake MOD movement (wei)	68	Mean movement (WD)	52	Sex	76	Std. of SPT dur. (WE)	32
	Nightwake IN movement (wei)	52	Std. of SIBD dur. (WE)	24	Sleep eff. trend	56	Movement in L5 h (WE)	44
	Std. of wake time (WD)	48	Std. of SIBD dur. (WD)	40	Std. of temp. mean	60	Temp. min–max diff.	36

## Data Availability

Not applicable.

## Supplementary Materials

The following supporting information can be downloaded at: https://www.mdpi.com/article/10.3390/s23031069/s1, Source Code S1: GGIR configuration and Logistic Regression Model Parameters. Table S1: Spearman correlation matrix between all features used in this study.
