# Supplementary material for: New Insights into Stroke from Continuous Passively Collected Temperature and Sleep Data Using Wrist-Worn Wearables
Source: Sensors (Basel). 2023 Jan 17;23(3):1069. doi: 10.3390/s23031069 (PMC9920931; doi:10.3390/s23031069)
Supplement: Supplementary file 1 [file sensors-23-01069-s001.zip › Source_Code_S1.pdf]

Section S1. GGIR configuration: Version 1.9-2

```
g.shell.GGIR(#-------------------------------
        # General parameters
        #-------------------------------
        mode=c(1,2,3,4,5),
        datadir=datadir,
        outputdir=outputdir,
        f0=0,
        f1=1,
        desiredtz = "Europe/London",
        overwrite = TRUE,
        print.filename=FALSE,
        storefolderstructure = FALSE,
        #-------------------------------
        # Part 1 parameters:
        #-------------------------------
        #-------------------------------
        # Part 2 parameters:
        #-------------------------------
        strategy = 1,
        # hrs.del.start = 0,
        # hrs.del.end = 0,
        maxdur = 60,
        includedaycrit = 16,
        #-------------------------------
        # Part 3 parameters:
        #-------------------------------
        ignorenonwear = FALSE, # this is left as FALSE as otherwise
        # nights with nonwear are set to 0 sleep and included in calculations
        #-------------------------------
        # Part 4 parameters:
        #-------------------------------
        excludefirstlast = FALSE,
        do.visual = TRUE,
        nnights = c(),
        def.noc.sleep = 1,  # uses HDCZA algorithm to detect SPT time in "sleeplog" variables
        relyonsleeplog= FALSE,
        includenightcrit = 16,
        #-----------------------------------
        # Report generation
        #-------------------------------
        do.report=c(2,4,5),
        visualreport = TRUE)
```

Code to install the version of GGIR in R:

```
# Using Version 1.9-2 of GGIR
require(devtools)
install_version("GGIR", version = "1.9.2", repos = "http://cran.us.r-project.org")
library(GGIR)
```

Section S2. Logistic Regression Model Parameters

From Scikit-Learn package, we implemented the logistic regression models using the linear_model.LogisticRegression command with the following parameters:

LogisticRegression(*max_iter=1000,*
# with the following default parameters:
penalty='l2', *dual=False*, *tol=0.0001*, *C=1.0*, *fit_intercept=True*, *intercept_scaling=1*, *class_weight=None*, *random_state=None*, *solver='lbfgs'*, *multi_class='auto'*, *verbose=0*, *warm_start=False*, *n_jobs=None*, *l1_ratio=None*)
